# Psychological Safety as a Mediator of the Relationship Between Inclusive Leadership and Nurse Voice Behaviors and Error Reporting

**DOI:** 10.1111/jnu.12689

**Published:** 2021-07-26

**Authors:** Seung Eun Lee, V. Susan Dahinten

**Affiliations:** ^1^ *Lambda Alpha at‐Large*, Assistant Professor, College of Nursing Yonsei University Seoul South Korea; ^2^ Associate Professor, School of Nursing University of British Columbia Vancouver BC Canada

**Keywords:** employee voice, error reporting intention, inclusive leadership, mediation analysis, psychological safety, speaking up, withholding voice

## Abstract

**Purpose:**

The purpose of this study was to examine psychological safety as a mediator of the relationship between inclusive leadership and nurses’ voice behaviors and error reporting. Voice behaviors were conceptualized as speaking up and withholding voice.

**Design:**

This correlational study used a web‐based survey to obtain data from 526 nurses from the medical/surgical units of three tertiary general hospitals located in two cities in South Korea.

**Methods:**

We used model 4 of Hayes’ PROCESS macro in SPSS to examine whether the effect of inclusive leadership on the three outcome variables was mediated by psychological safety.

**Findings:**

Mediation analysis showed significant direct and indirect effects of nurse managers’ inclusive leadership on each of the three outcome variables through psychological safety after controlling for participant age and unit tenure. Our results also support the conceptualization of employee voice behavior as two distinct concepts: speaking up and withholding voice.

**Conclusions:**

When leader inclusiveness helps nurses to feel psychologically safe, they are less likely to feel silenced, and more likely to speak up freely to contribute ideas and disclose errors for the purpose of improving patient safety.

**Clinical Relevance:**

Leader inclusiveness would be especially beneficial in environments where offering suggestions, raising concerns, asking questions, reporting errors, or disagreeing with those in more senior positions is discouraged or considered culturally inappropriate.

Patient safety is an international priority. Since the 2000 publication of *To Err is Human* by the Institute of Medicine (IOM), significant improvements have been made in the safety of health care around the world. However, a large number of patients are still harmed by preventable errors while receiving health‐care services (World Health Organization, 2019). For example, medical errors remain the third leading cause of patient death in the United States (Makary & Daniel, 2016) and Canada (RiskAnalytica, 2017). In South Korea, the extent of the problem cannot even be accurately estimated due to the limited reporting of medical errors. Following establishment of the Patient Safety Act of 2016, the Ministry of Health and Welfare (MOHW) set up a voluntary error reporting system but reporting seems to have been incomplete and the Korean government is now considering mandatory error reporting (MOHW, 2020).

Error reporting is fundamental to reducing patient errors and improving patient safety, but equally important is the willingness of staff to speak up proactively to improve patient safety. In Korea, one potential reason for the shortage of data on medical error is a hesitation to report errors, and this may be coupled with a hesitation to communicate concerns or suggestions for the purpose of improving patient safety. The latter has been referred to as *employee voice behavior*, comprising a continuum from silence to voice (Park & Kim, 2016), although other authors have conceptualized employee silence as a construct that is distinct from employee voice (i.e., van Dyne, Ang, & Botero, 2003). Schwappach and Richard (2018) used the terms *speaking up* and *withholding voice* to refer to employee voice and employee silence, respectively.

Due to their frontline role in providing patient care, nurses have been recognized as key contributors to the improvement of patient safety (IOM, 2003). Although nurses’ voice and error reporting behaviors are crucial for improving patient safety, remaining silent and underreporting of errors are well‐recognized phenomena in health care (Schwappach & Richard, 2018; Soydemir, Seren Intepeler, & Mert, 2017). For Korean nurses, speaking up and reporting errors may be particularly difficult because Korean culture, like many other Asian cultures, does not encourage open communication in regard to social and organizational problems. Rather, the culture emphasizes collectivism, obedience, and respect for authority (Ishikawa & Yamazaki, 2005; Pun, Chan, Wang, & Slade, 2018). In such a culture, nurses often do not feel safe making suggestions, asking questions, or reporting something that may negatively influence patient care (Lee et al., 2019). In Korean culture, employees in all occupations are more likely to keep silent rather than speak up because they value interdependence, harmony, conformance with social expectations, and conflict avoidance in the workplace (Park & Kim, 2016). Similarly, nurses in Korean health‐care settings may be more likely to withhold their voices than voice their ideas or concerns, or report their own or others’ errors.

One promising avenue for improving the voice behavior and error reporting of nurses is establishing a state of psychological safety (Alingh, van Wijngaarden, van de Voorde, Paauwe, & Huijsman, 2019; Edmondson, 2018), defined as “a shared belief that the team is safe for interpersonal risk taking” (Edmondson, 1999, p. 354). Employees are more likely to speak up and report errors when they do not fear rejection, disapproval, or blame from others regarding their ideas, concerns, and errors (Appelbaum, Dow, Mazmanian, Jundt, & Appelbaum, 2016; Edmondson, 2018; Nembhard & Edmondson, 2006). Previous research has identified psychological safety as a significant antecedent to employee voice behavior and error reporting in organizations (Appelbaum et al., 2016; Edmondson & Lei, 2014; O'Donovan & McAuliffe, 2020; Pfeiffer, Manser, & Wehner, 2010).

Organizational literature has highlighted the importance of leadership to psychological safety and employee voice behaviors (Edmondson & Lei, 2014; Edmondson, 2018; Morrison, 2014; Nembhard & Edmondson, 2006). For example, a review of 11 qualitative studies found that open and supportive leadership could foster the speaking‐up behaviors of health‐care workers by creating an environment where it is safe to speak up (Morrow, Gustavson, & Jones, 2016). One emerging leadership style that could positively impact patient safety is inclusive leadership (Amin, Till, & McKimm, 2018), which is characterized by a clear exhibition of openness, availability, and accessibility (Carmeli, Reiter‐Palmon, & Ziv, 2010). Inclusive leaders actively invite others to voice their opinions and provide input, and show appreciation for those contributions; thus, staff feel that their voices are heard and valued (Nembhard & Edmondson, 2006). For example, Carmeli et al. (2010) demonstrated that inclusive leadership led to increased psychological safety, which in turn resulted in greater engagement in creative work tasks among staff in the technology industry. In health care, leader inclusiveness could similarly increase psychological safety in the workplace and decrease nurses’ fear of potentially negative consequences from speaking up and error reporting behaviors. To the best of our knowledge, only one study has tested psychological safety as a mediator of inclusive leadership (Appelbaum et al., 2016), but the outcome was limited to error reporting among medical residents. Thus, there is a gap in our knowledge regarding the effects of inclusive leadership and psychological safety among nurses, and with respect to speaking up and withholding voice.

This study aimed to investigate the relationship among inclusive leadership, psychological safety, and three nurse outcomes—speaking up, withholding voice, and error reporting intention. We hypothesized that (1) inclusive leadership is positively associated with psychological safety, (2) psychological safety is positively associated with speaking up and error reporting intention and negatively associated with withholding voice, and (3) psychological safety mediates the association between inclusive leadership and the three outcomes.

## METHODS

### Setting and Sample

We employed a correlational study design with cross‐sectional survey data from nurses working in the medical or surgical units of three tertiary general hospitals located in two cities in South Korea. Tertiary general hospitals have 500+ beds, and provide advanced medical services to critically ill patients. Also, to promote sample homogeneity, we included only nurses involved in direct patient care. Following Fritz and MacKinnon’s (2007) guidance for sample size requirements in mediation analysis using the percentile bootstrap method, we determined that 412 cases were required to achieve 0.8 power for a small effect of a predictor on a mediator, and small‐to‐medium effect of the mediator on an outcome variable, controlling for the mediator.

On behalf of the research team, the hospitals sent out invitation emails with a secure link to the web‐based survey to 731 nurses working in medical/surgical units. Two reminder emails were sent out 2 and 3 weeks after the first invitation. A total of 526 nurses completed the survey, yielding a response rate of 72%. A gift certificate was offered as honorarium for participation (equivalent to US$9). The study protocol was approved by the institutional review board of Yonsei university health system (Y‐2020‐0013), and data were collected in May and June 2020.

### Measures

The survey included five scales that measured inclusive leadership, psychological safety, speaking up, withholding voice, and nurses’ intention to report errors. The survey also collected demographic information on the nurses’ age, gender, education level, employment status, years of nursing experience, unit tenure, and hospital tenure. All scales except for nurses’ intention to report errors were translated into Korean as part of this study using a committee approach to translation (Furukawa et al., 2014). The committee was comprised of four bilingual Korean health professionals (three nursing professors and one hospital nurse) who were familiar with the health‐care environments of both the United States and Korea. The four committee members translated each scale independently, and then came to a consensus on the final version of each scale. The translated instruments were then reviewed for content validity (cultural relevance and appropriateness) by an expert panel consisting of 10 patient safety experts working in academic and clinical settings. As a final step in the translation process, the instruments were pilot‐tested with 10 Korean nurses who worked on medical/surgical units to confirm the clarity and readability of the translated items, response options, and survey instructions.

The perceptions of their manager’s inclusive leadership were measured with a nine‐item instrument developed by Carmeli et al. (2010). Although this instrument assesses three dimensions of inclusive leadership—openness (three items), availability (four items), and accessibility (two items)—Carmeli et al. found that the nine items reflected a single factor. Responses were measured on a five‐point scale ranging from 1 (*not at all*) to 5 (*to a large extent*). For the study sample, exploratory factor analysis with principle component analysis supported the one‐factor model, which explained 69.6% of the variance; factor loadings ranged from 0.80 to 0.86.

Psychological safety was measured using a seven‐item measure developed by Edmondson (1999), and a five‐point response scale ranging from 1 (*strongly disagree*) to 5 (*strongly agree*). In a previous study, this measure demonstrated content, criterion, and construct validity (Newman, Donohue, & Eva, 2017). For the study sample, exploratory factor analysis with principle component analysis yielded a one‐factor model, which explained 42.0% of the variance; factor loadings ranged from 0.57 to 0.68.

Speaking up was measured with four items from the Korean version of the Hospital Survey on Patient Safety Culture 2.0 (Lee & Dahinten, 2021). The items asked respondents’ perceptions of their own and their unit’s frequency of speaking up regarding patient safety (Sorra, Yount, Famolaro, & Gray, 2019). Responses were measured on a five‐point scale ranging from 1 (*never*) to 5 (*always*); one negatively worded item was reverse‐scored.

Withholding voice was measured using four items from the Speaking Up about Patient Safety Questionnaire (Richard, Pfeiffer, & Schwappach, 2017). The items assess the frequency of choosing not to speak up in particular types of situations over the previous 4 weeks. Responses are measured on a five‐point Likert scale ranging from 1 (*never*) to 5 (*very often*, more than 11 times). Exploratory factor analysis with principle component analysis supported the one‐factor model, which explained 77.8% of the variance; loadings ranged from 0.84 to 0.93.

Nurses’ intention to report their own or others’ errors was measured using a three‐item scale developed by Kim (2010). Responses indicate error reporting intention on a five‐point scale ranging from 1 (*never*) to 5 (*always*). In previous studies, the scale’s Cronbach’s *α* ranged from 0.83 to 0.85 with a Korean nurse population (Kim, 2010; Ko & Yu, 2017). For the study sample, exploratory factor analysis with principle component analysis supported a one‐factor model, which explained 72.47% of the variance; factor loadings ranged from 0.74 to 0.91.

For each of the five scales, total mean scores were computed, with higher scores indicating a higher level of the construct. Cronbach’s *α* for the five scales ranged from 0.73 to 0.95, and are reported for each scale in Table 1.

**TABLE 1:** Correlations, descriptive statistics, and Cronbach’s *α* for key study variables

Variable	1	2	3	4	5
Inclusive leadership	—	—	—	—	—
Psychological safety	0.53***	—	—	—	—
Speaking up	0.50***	0.52***	—	—	—
Withholding voice	−0.21***	−0.26***	−0.28***	—	—
Error reporting intention	0.23***	0.23***	0.32***	−0.18***	—
M	3.52	3.35	3.23	1.81	3.57
SD	0.68	0.51	0.58	0.68	0.70
Cronbach’s *α*	0.95	0.76	0.73	0.90	0.81

Note.

*N* = 526, ***p < 0.001.

### Statistical Analysis

Data were analyzed using IBM SPSS 25.0 and PROCESS macro version 3.5 for SPSS with the statistical significance level set at *p* < 0.05. Descriptive statistics were used to describe sample characteristics and study variables. Relationships between study variables were examined using Pearson correlation analysis. We used model 4 of the PROCESS macro in SPSS (Hayes, 2017) to examine whether the effect of inclusive leadership on each outcome variable was mediated by psychological safety. Based on previous research (Alingh et al., 2019; Omura, Stone, & Levett‐Jones, 2018), we controlled for participant age and unit tenure in this analysis due to their probable relationship with the outcome variables. The statistical significance of the indirect mediation effect on each outcome variable was assessed by bootstrapping (5000 samples) with a 95% confidence interval (Hayes, 2017).

## RESULTS

The 526 study participants were predominantly (98.3%) female with a mean age of 31.2 years (SD = 11.3) and 7.5 years (SD = 6.5) of nursing experience. Their mean unit tenure was 4.4 years (SD = 3.8) and mean hospital tenure was 7.1 years (SD = 6.5). Almost all participants (99.4%) had a permanent, full‐time position, and 95% had a bachelor’s or higher degree in nursing.

Descriptive statistics and intercorrelations between key study variables are presented in Table 1. Bivariate correlation results showed that nurse managers’ inclusive leadership was positively associated with nurses’ psychological safety, speaking up, and error reporting intention, and negatively associated with withholding voice. Also, nurses’ psychological safety was positively related to speaking up and error reporting intention and negatively related to withholding voice. Notably, among the outcome variables, speaking up and withholding voice showed a low correlation of −0.28 (*p* < 0.001), and speaking up and error reporting intention yielded a correlation of 0.32 (*p* < 0.001).

### Mediation Analysis

**FIGURE 1:**
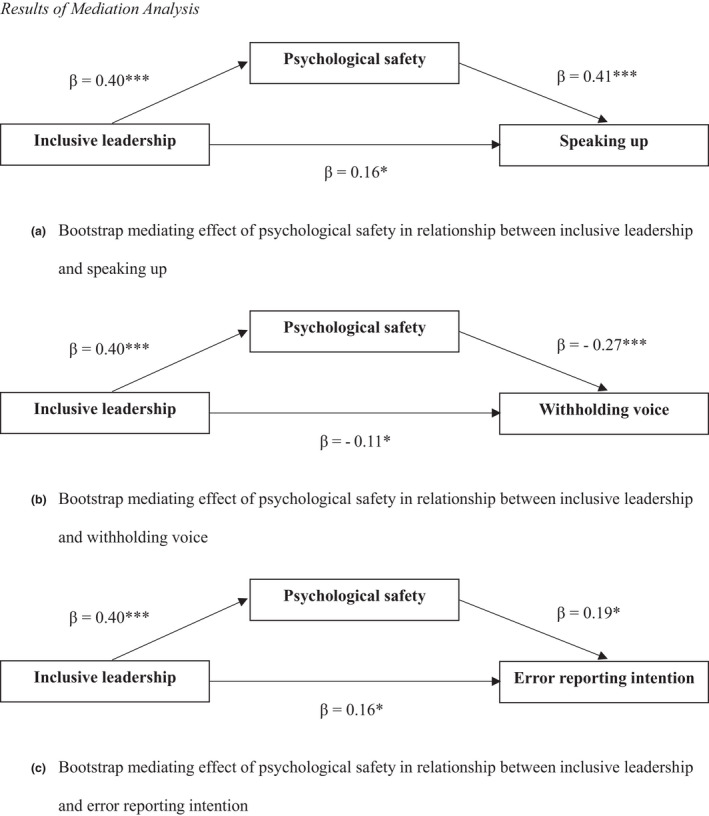
Results of mediation analysis. Bootstrap mediating effect of psychological safety in relationship between (a) inclusive leadership and speaking up, (b) inclusive leadership and withholding voice, and (c) inclusive leadership and error reporting intention. *Note*. All models were controlled for participant age and unit tenure. *p < 0.05, **p < 0.01, ***p < 0.001

As illustrated in Figure 1, the mediation analysis results showed a significant direct and indirect effect of nurse managers’ inclusive leadership on each of the three outcome variables through psychological safety while controlling for participant age and unit tenure. Specifically, inclusive leadership was positively associated with psychological safety (*B* = 0.40, SE = 0.03, *p* < 0.001), and psychological safety was significantly related to speaking up (*B* = 0.41, SE = 0.05, *p* < 0.001), withholding voice (*B* = −0.27, SE = 0.07, *p* < 0.001), and error reporting intention (*B* = 0.19, SE = 0.07, *p* < 0.01). The association between inclusive leadership and the three outcome variables remained significant after controlling for psychological safety, indicating that psychological safety only partially mediated the effect of inclusive leadership on speaking up (*B* = 0.16, SE = 0.04, 95% CI = 0.12 to 0.21), withholding voice (*B* = −0.11, SE = 0.05, 95% CI = −0.16 to −0.05), and error reporting intention (*B* = 0.08, SE = 0.03, 95% CI = 0.01–0.15).

## DISCUSSION

To our knowledge, this study is the first to examine the relationships among inclusive leadership, psychological safety, and speaking up, withholding voice, and the error reporting intentions of health‐care professionals. Thus, this study contributes to the scarce literature examining inclusive leadership and employee silence and voice in general (Guo, Zhu, & Zhang, 2020). Also, examination of the role of psychological safety in the relationship between inclusive leadership and the three outcomes in the Korean context was valuable, as previous research into the psychological safety of employees in work settings has been mainly conducted in Western cultures (Newman et al., 2017). Overall, our study showed that inclusive leadership on the part of nurse managers was positively related to nurses’ psychological safety, which in turn was associated with higher levels of speaking up and error reporting intention as well as lower levels of withholding voice among medical/surgical nurses in Korean hospitals. Our study findings indicated that psychological safety played a mediating role in the relationship between inclusive leadership and the three nurse outcomes. Finally, our results also support the conceptualization of employee voice behavior as two distinct concepts—speaking up and withhold voice.

Our findings show the importance of inclusive leadership to nurses’ speaking up and withholding voice behaviors, and their error reporting intentions. Inclusive leaders are characterized as being open, accessible, and available to their staff (Carmeli et al., 2010). Our findings imply that when nurses perceive that their managers are open to suggestions for improving work processes to achieve better patient outcomes, and are willing to have interactive relationships with them, the nurses feel safe to speak up rather than withholding their voice, and are more willing to report errors (Carmeli et al., 2010; Nembhard & Edmondson, 2006). The limited previous research has shown similar results. For example, leader inclusiveness was positively associated with voice behaviors of Chinese employees (Guo et al., 2020) and with American medical residents’ intention to report patient adverse events (Appelbaum et al., 2016). Thus, nurse managers should strive to exhibit behaviors that are inclusive of their subordinates, such as sharing critical information, initiating meetings to discuss the progress of patients, being available for consultation, and maintaining an ongoing presence within the team (Appelbaum et al., 2016; Bowers, Robertson, & Parchman, 2012; Carmeli et al., 2010). These types of leadership behaviors give nurses opportunities to ask questions and discuss patient safety concerns, and they establish a climate of psychological safety in which nurses feel encouraged to report errors.

Inclusive leaders create a culture of psychological safety by valuing and respecting their staff’s views even when those views conflict with their own (Bowers et al., 2012; Guo et al., 2020). Also, where higher status staff may already experience a degree of psychological safety, inclusive leaders help staff of lower status feel psychologically safe (Derickson, Fishman, Osatuke, Teclaw, & Ramsel, 2015) by assuring them that no negative consequences will result from voicing concerns or reporting errors. A psychologically safe environment supports learning from mistakes so that staff will be open to providing feedback, challenging the system, and contributing new ideas (Amin et al., 2018), which in turn can result in positive workplace outcomes. Consistent with previous research (Carmeli et al., 2010; Guo et al., 2020; Nembhard & Edmondson, 2006), our findings highlight the importance of leader inclusiveness to the development of psychological safety among subordinates. Thus, nurse managers are well advised to exhibit inclusive leadership to their staff in order to create an environment in which nurses feel psychologically safe to speak up and to report errors.

This study demonstrated that nurses’ psychological safety was positively associated with speaking up and error reporting intention, and negatively related to withholding voice. Our findings extend understanding of how inclusive leadership can alleviate voice withholding and encourage nurses to speak up and to report errors through the establishment of a psychologically safe workplace (Alingh et al., 2019; Appelbaum et al., 2016). In addition, the low correlations we found between the three outcome variables suggest that speaking up and withholding voice are distinct concepts (Schwappach & Richard, 2018) rather than opposite ends of a continuum, and that speaking up and error reporting are also different. We speculate that speaking up and error reporting may yield different ultimate outcomes; speaking up might contribute to improvements in quality of care that go beyond patient safety, which is just one of the components of healthcare quality (Lee et al., 2019). Future research is needed to confirm these findings.

In many health‐care contexts, fear of speaking up and error reporting exists due to an unhealthy organizational culture, which deprives nurses and other healthcare providers of the opportunity to contribute to improved patient safety and quality of care. Especially in Asian cultures and organizations where collectivism, hierarchy, seniority, and obedience are emphasized, nurses often feel uncomfortable raising their voice and disclosing errors, even when they observe situations that may adversely influence patient care (Lee & Quinn, 2020). Thus, in cultures such as Korea’s, nurses’ psychological safety could play a central role in their voice behaviors and error reporting. In previous studies, Korean nurses have perceived their work culture as punitive and reported that they would not speak up or ask questions in their workplace (Kim, Kim, Lee, & Oh, 2018). As inclusive leadership behaviors have been shown to be key to developing subordinates’ psychological safety (Appelbaum et al., 2016; Edmondson & Lei, 2014), the promotion of such behaviors among clinical leaders is a logical strategy for developing psychological safety in nurses. Therefore, nurse managers should proactively foster open communication with their staff and show a willingness to listen and respond to staff concerns, ideas, and recommendations, for the sake of improving patient safety (Lee et al., 2018). When nurses perceive that speaking up and reporting errors are not risky behaviors, but rather, are welcomed, they will be more likely to disclose errors, challenge the status quo, and offer ideas to enhance patient safety and quality of care.

## LIMITATIONS

Some limitations of this study should be noted. First, data were collected using a cross‐sectional design, which precludes causal inference. Second, all data were collected using self‐report questionnaires, and thus may have been subject to self‐reporting bias. Third, our sample was limited to nurses working in medical/surgical units in Korean tertiary general hospitals, and our participants were predominantly female nurses working full time; thus, the findings of this study should be generalized with caution. Future studies should examine the relationship among inclusive leadership, psychological safety, and error reporting in more diverse cultural and organizational contexts. Finally, although we controlled for participant age and unit tenure in our analyses, unmeasured or unknown factors such as communication skills, specific educational background, hospital policy, and organizational culture (Okuyama, Wagner, & Bijnen, 2014; Soydemir et al., 2017) could have contributed to the associations found in this study.

## CONCLUSIONS

This study provides initial evidence that nurses’ voice behaviors and error reporting intentions are associated with their perceptions of psychological safety, which are positively related to inclusive leadership. Thus, this study contributes to the scant research examining these relationships in the nursing profession. Our findings show that by exhibiting inclusive behaviors, nurse managers could encourage their staff to speak up rather than remaining silent, and to report errors. Leader inclusiveness would be especially beneficial in environments where offering ideas or suggestions, asking questions, providing feedback, raising concerns, or disagreeing with those having more authority and seniority is considered culturally inappropriate or even unacceptable. When leader inclusiveness helps nurses to feel psychological safe, they will speak up freely, disclose errors, thus creating more opportunities to improve patient safety.
